# FKBP12-Dependent Inhibition of Calcineurin Mediates Immunosuppressive Antifungal Drug Action in *Malassezia*

**DOI:** 10.1128/mBio.01752-17

**Published:** 2017-10-24

**Authors:** Giuseppe Ianiri, Shelly Applen Clancey, Soo Chan Lee, Joseph Heitman

**Affiliations:** a Department of Molecular Genetics and Microbiology, Duke University Medical Center, Durham, North Carolina, USA; b Department of Biology, South Texas Center for Emerging Infectious Diseases (STCEID), The University of Texas at San Antonio, San Antonio, Texas, USA; Friedrich Schiller University Jena

**Keywords:** FKBP12-binding protein, *Malassezia*, calcineurin inhibitors, hypermutator

## Abstract

The genus *Malassezia* includes yeasts that are commonly found on the skin or hair of animals and humans as commensals and are associated with a number of skin disorders. We have previously developed an *Agrobacterium tumefaciens* transformation system effective for both targeted gene deletion and insertional mutagenesis in *Malassezia furfur* and *M. sympodialis*. In the present study, these molecular resources were applied to characterize the immunophilin FKBP12 as the target of tacrolimus (FK506), ascomycin, and pimecrolimus, which are calcineurin inhibitors that are used as alternatives to corticosteroids in the treatment of inflammatory skin disorders such as those associated with *Malassezia* species. While *M. furfur* and *M. sympodialis* showed *in vitro* sensitivity to these agents, *fkb1*Δ mutants displayed full resistance to all three of them, confirming that FKBP12 is the target of these calcineurin inhibitors and is essential for their activity. We found that calcineurin inhibitors act additively with fluconazole through an FKBP12-dependent mechanism. Spontaneous *M. sympodialis* isolates resistant to calcineurin inhibitors had mutations in the gene encoding FKBP12 in regions predicted to affect the interactions between FKBP12 and FK506 based on structural modeling. Due to the presence of homopolymer nucleotide repeats in the gene encoding FKBP12, an *msh2*Δ hypermutator of *M. sympodialis* was engineered and exhibited an increase of more than 20-fold in the rate of emergence of resistance to FK506 compared to that of the wild-type strain, with the majority of the mutations found in these repeats.

## IMPORTANCE

*Malassezia* species are the most abundant fungal components of the mammalian and human skin microbiome. Although they belong to the natural skin commensal flora of humans, they are also associated with a variety of clinical skin disorders. The standard treatment for *Malassezia*-associated inflammatory skin infections is topical corticosteroids, although their use has adverse side effects and is not recommended for long treatment periods. Calcineurin inhibitors have been proposed as a suitable alternative to treat patients affected by skin lesions caused by *Malassezia*. Although calcineurin inhibitors are well-known as immunosuppressive drugs, they are also characterized by potent antimicrobial activity. In the present study, we investigated the mechanism of action of FK506 (tacrolimus), ascomycin (FK520), and pimecrolimus in *M. furfur* and *M. sympodialis* and found that the conserved immunophilin FKBP12 is the target of these drugs with which it forms a complex that directly binds calcineurin and inhibits its signaling activity. We found that FKBP12 is also required for the additive activity of calcineurin inhibitors with fluconazole. Furthermore, the increasing natural occurrence in fungal pathogen populations of mutator strains poses a high risk for the rapid emergence of drug resistance and adaptation to host defense. This led us to generate an engineered hypermutator *msh2*Δ mutant strain of *M. sympodialis* and genetically evaluate mutational events resulting in a substantially increased rate of resistance to FK506 compared to that of the wild type. Our study paves the way for the novel clinical use of calcineurin inhibitors with lower immunosuppressive activity that could be used clinically to treat a broad range of fungal infections, including skin disorders caused by *Malassezia*.

## INTRODUCTION

The *Malassezia* genus includes a monophyletic group of yeasts within the basidiomycetes. Compared to related saprophytes and fungal pathogens of plants and mammals, *Malassezia* has a commensal lifestyle on the skin and hair of animals and humans, and represents the most abundant fungal component of the human skin microbiome (1–4). The distribution of *Malassezia* species in different body sites differs between individuals and is influenced by age, but in general, the most common species on human skin are *Malassezia  globosa*, *M. restricta*, and *M. sympodialis*. Other species, such as *M. furfur*, are rarely isolated as a commensal but have been reported as fungemia-causing species as a consequence of catheter-associated fungal growth and lipid-rich parenteral nutrition (5–7). The number of described species within the *Malassezia* genus has increased (8, 9), and at the present, 17 species have been reported. In recent metagenomic studies, *Malassezia* DNA has been detected in the lungs and on mucosal surfaces of patients (3), but also in environmental samples (10), indicating that this fungal genus is still poorly understood and that new species may remain to be described.

The last decade of genomic research on *Malassezia* has highlighted its unique features among fungi that likely reflect the evolutionary trajectory of this genus’s association with restricted ecological niches. *Malassezia* species have small genomes (from ~7 to ~9 Mbp), with the exception of a few hybrid strains of *M. furfur* that have twice the size of other *M. furfur* genomes. Interestingly, *Malassezia* species have lost fatty acid synthetase genes and are hence dependent on lipids, relying on a number of secreted lipase and phospholipase families that are highly represented in their genomes, likely to utilize extracellular lipids for growth. The presence of unique gene families, the acquisition of bacterial genes through horizontal gene transfer (HGT), and extensive loss of hydrolases and other genes involved in carbohydrate metabolism are other features of *Malassezia* genomes (11–13).

Although *Malassezia* yeasts are normal skin commensals, they have been found in association with a variety of clinical skin disorders, the most common of which are pityriasis versicolor, dandruff, psoriasis, atopic and seborrheic dermatitis, and folliculitis. There are studies in which *Malassezia* yeasts have been found in the lungs of patients with cystic fibrosis and in the brains of patients diagnosed with Alzheimer’s disease, and other studies that reported systemic *Malassezia* infections in patients receiving total parenteral nutrition or in immunocompromised hosts (1, 14–16).

Despite the importance of the *Malassezia* yeasts, genetic and biochemical mechanisms that underlie their dual lifestyle as a commensal and a pathogen, as well as interactions with the host, have not been extensively investigated. This lack of research has resulted in controversial hypotheses on the exact role of *Malassezia* in these clinical conditions (17, 18). For example, in inflammatory conditions (i.e., seborrheic and atopic dermatitis), *Malassezia* is considered an exacerbating factor that acts as an allergen rather than as an infective agent, a hypothesis supported by the reactivity of allergen-specific IgE and T cells in sera and blood from patients (19). Moreover, a set of 13 *Malassezia* allergens have been identified; some of these have unknown functions and no known orthologs, while others share homology with host proteins, suggesting the possibility of cross-reactivity with the immune response (18, 19).

Localized skin diseases caused by *Malassezia* are treated with topical azoles, while extensive skin diseases as well as disseminated infections require systemic administration of amphotericin B or azoles (14, 15). *Malassezia*-associated inflammatory conditions require instead anti-inflammatory therapy that has been based for decades on the use of corticosteroids (15, 19). While these agents are of proven efficacy in reducing disease symptoms, long-term treatment is not recommended because they are associated with a number of side effects. Moreover, considering that children represent the majority of the patient population affected by inflammatory dermatitis, the use of corticosteroids is highly discouraged (20). Topical calcineurin inhibitors, including tacrolimus (FK506, Protopic) and pimecrolimus (Elidel), have been introduced recently as a suitable alternative for treating inflammatory skin lesions in patients who do not respond to classical therapy (20). New formulations based on nano-size carrier particles that enhance FK506 penetration through the skin and increase its efficacy have also been developed (21). However, a few years after the introduction of topical calcineurin inhibitors, the U.S. Food and Drug Administration issued a “black box” warning for a theoretical risk of lymphoma associated with their use (22). At present, this warning still remains, and despite the number of studies that demonstrate no correlation with malignancy, a critical review by Siegfried and colleagues highlighted the lack of information for long-term therapy based on calcineurin inhibitors, and hence, their use for inflammatory dermatitis is recommended for only intermittent periods of time (20).

The mechanism of action of calcineurin inhibitors has been extensively investigated, revealing that these drugs bind to peptidyl-propyl *cis-trans* isomerase immunophilins—FK506 binds FKBP12, and cyclosporine binds cyclophilin A—to form complexes that bind calcineurin and inhibit its activity (23). While in humans this binding hinders the immune response, allowing the use of calcineurin inhibitors to prevent organ rejection in transplant recipients, in fungi these agents also inhibit fungal calcineurin, which is involved in a number of cellular functions (responses to stress, cell wall integrity, resistance to antifungal drugs, and virulence) and thus represents an attractive target for antifungal drug development (24, 25). The antifungal activity of FK506 (tacrolimus) and pimecrolimus for several *Malassezia* species has been previously reported (26, 27), leading to the hypothesis of a dual action of these molecules that suppress host immune response and at the same time inhibit the growth of the fungus that triggers the host immune responses (19).

In this study, we investigated the mechanisms of action of calcineurin inhibitors in *M. furfur* and *M. sympodialis*. We demonstrate the following. (i) Given the effects of FK506, we conclude that calcineurin in *M. furfur* and *M. sympodialis* is essential for growth at elevated temperature, plasma membrane functionality, and cation homeostasis. (ii) Disruption of the *FKB1* gene encoding FKBP12 confers resistance to FK506, pimecrolimus, and ascomycin in both *M. furfur* and *M. sympodialis*. (iii) Calcineurin inhibitors act additively with fluconazole (FLC) in an FKBP12-dependent manner. (iv) Spontaneous resistance to calcineurin inhibitors arises through mutations in the gene encoding FKBP12 that result in the production of FKBP12 with altered conformational structures or altered ability to interact with FK506, as predicted following modeling simulations. (v) An *msh2*Δ hypermutator of *M. sympodialis* shows a much higher rate of resistance to FK506 compared to the wild type (WT), and this is due to mutations in homopolymer repeats in the gene encoding FKBP12 that result in a premature stop codon.

## RESULTS

### Calcineurin inhibitors display antifungal activity against *M. furfur* and *M. sympodialis*.

**FIG 1:**
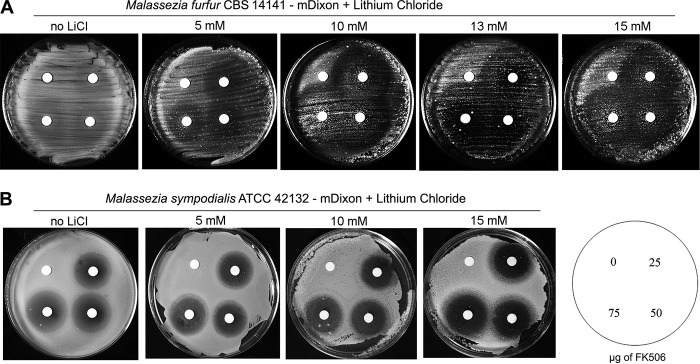
The calcineurin inhibitor FK506 displays antifungal activity against *M. furfur* and *M. sympodialis*. (A and B) Cells of *M. furfur* CBS 14141 and *M. sympodialis* ATCC 42132 were grown on mDixon medium alone or supplemented with 5 mM, 10 mM, 13 mM (only for *M. furfur*), or 15 mM LiCl and exposed to disks containing 0 (vehicle [DMSO]), 25 µg, 50 µg, and 75 µg of FK506.

Initially, FK506 was used as a test drug to elucidate the conditions under which calcineurin could be essential in several strains of *M. furfur* and *M. sympodialis* (Table 1). Antifungal susceptibility disk diffusion assays were performed on modified potato dextrose agar (mPDA) and modified Dixon’s medium (mDixon) (5) at 30°C and 37°C, because these conditions were previously shown to support growth of several strains of these two *Malassezia* species (28). FK506 had no effect on the growth of *M. furfur* CBS 14141 on mPDA (data not shown), and it displayed only marginal toxicity when assays were performed on mDixon medium. The addition of LiCl, which was found to be critical for calcineurin inhibitor toxicity against *Saccharomyces cerevisiae* (29), was found to be essential also for antifungal activity of FK506 against *M. furfur* CBS 14141, with growth inhibition paralleling the concentration increase of LiCl, achieving the strongest toxicity at 13 mM (Fig. 1A). When incubation was performed at 30°C, FK506 displayed no antifungal activity against *M. furfur*; also, *M. furfur* CBS 7982 showed no clear sensitivity to FK506 under any of the conditions tested (data not shown). FK506 showed antifungal activity against *M. sympodialis* strains ATCC 42132 and ATCC 44340 at 37°C even on mDixon agar alone, although the addition of LiCl up to 15 mM increased FK506 toxicity (Fig. 1B; also see Fig. S1 in the supplemental material). With experiments performed at 30°C, the antifungal activity of FK506 was drastically reduced, even in the presence of LiCl (Fig. S2), indicating that high temperature is crucial for the activity of FK506 and thus for calcineurin function. For the type strain CBS 7222 of *M. sympodialis*, a temperature of 37°C and 15 mM LiCl in mPDA medium were essential conditions for clear toxicity of FK506 (Fig. S3).

10.1128/mBio.01752-17.1 FIG S1  Effect of LiCl on the antifungal activity of FK506 against *M. sympodialis* ATCC 44340 at 37°C. Cells were grown on mDixon medium supplemented with 5 mM or 15 mM LiCl and exposed to disks containing 0 (vehicle [DMSO]; top left disk), 25 µg (top right disk), 50 µg (bottom right disk), or 75 µg of FK506 (bottom left disk). Download FIG S1, TIF file, 1.5 MB. Copyright © 2017 Ianiri et al. 2017 Ianiri et al. This content is distributed under the terms of the Creative Commons Attribution 4.0 International license.

10.1128/mBio.01752-17.2 FIG S2  Antifungal activity of FK506 against *M. sympodialis* ATCC 42132 and ATCC 44340 at 30°C. Cells were grown on mDixon medium supplemented with 15 mM LiCl and exposed to disks containing 0 (vehicle), 25 µg, 50 µg, or 75 µg of FK506 as indicated in Fig. S1 in the supplemental material. Download FIG S2, TIF file, 1.4 MB. Copyright © 2017 Ianiri et al. 2017 Ianiri et al. This content is distributed under the terms of the Creative Commons Attribution 4.0 International license.

10.1128/mBio.01752-17.3 FIG S3  Effect of LiCl on the antifungal activity of FK506 against *M. sympodialis* CBS 7222 at 37°C. Cells were grown on mPDA medium supplemented with 5 mM or 15 mM LiCl and exposed to disks containing 0 (vehicle), 25 µg, 50 µg, or 75 µg of FK506 as indicated in Fig. S1. Download FIG S3, TIF file, 0.4 MB. Copyright © 2017 Ianiri et al. 2017 Ianiri et al. This content is distributed under the terms of the Creative Commons Attribution 4.0 International license.

**TABLE 1:** *Malassezia* strains used in the present study

*Malassezia* strain	Relevant genotype or change in the gene or protein	Background	Reference
*M. furfur* strains			
CBS 14141	Wild type		63
CBS 7982	Wild type		63
*fkb1*Δ mutant	*fkb1*Δ::*NAT*	*M. furfur* CBS 14141	This study

*M. sympodialis* strains			
ATCC 42132	Wild type		64
ATCC 44340	Wild type		64
CBS 7222	Wild type		64
*fkb1*Δ mutant	*fkb1*Δ::*NAT*	*M. sympodialis* ATCC 42132	This study
*msh2*Δ mutant	*msh2*Δ::*NAT*	*M. sympodialis* ATCC 42132	This study
SR1	*fkb1* 79T>G/FKBP12 Y27D	*M. sympodialis* ATCC 42132	This study
SR2	*fkb1* 116A>G/FKBP12 D39G	*M. sympodialis* ATCC 42132	This study
SR3	*fkb1* 227T>C/FKBP12 L76S	*M. sympodialis* ATCC 42132	This study
SR4	*fkb1* 232T>C/FKBP12 C78R	*M. sympodialis* ATCC 42132	This study
SR5	*fkb1* 296T>G/FKBP12 L99R	*M. sympodialis* ATCC 42132	This study
SR6	*fkb1* 314T>G/ FKBP12 L105R	*M. sympodialis* ATCC 42132	This study

Under the same conditions in which FK506 displayed the highest toxicity, the calcineurin inhibitors ascomycin and pimecrolimus also showed similar antifungal activity against both *M. furfur* CBS 14141 (see Fig. 3A) and *M. sympodialis* ATCC 42132 (see Fig. 4A). Two other drugs that target mTOR (mechanistic target of rapamycin) or calcineurin, rapamycin, and cyclosporine, respectively, showed no or low toxicity against the *M. furfur* and *M. sympodialis* strains used in the present study (data not shown).

For the following experiments, *M. furfur* CBS 14141 and *M. sympodialis* ATCC 42132 strains were utilized for several reasons. (i) They were the most sensitive to calcineurin inhibitors. (ii) Their genomes have been sequenced, with the annotation of *M. sympodialis* ATCC 42132 recently improved with proteogenomics (5, 12, 30). (iii) Effective systems for mutagenesis are available in both species (28).

### Mutation of the gene encoding FKBP12 of *M. furfur* and *M. sympodialis* confers resistance to calcineurin inhibitors.

Previous studies revealed that the conserved fungal protein FKBP12 is the target of the calcineurin inhibitor FK506 (24, 25). To validate this finding in *Malassezia*, we aimed to disrupt the gene encoding FKBP12 by homologous recombination in *M*. *furfur* CBS 14141 and *M. sympodialis* ATCC 42132.

**FIG 2:**
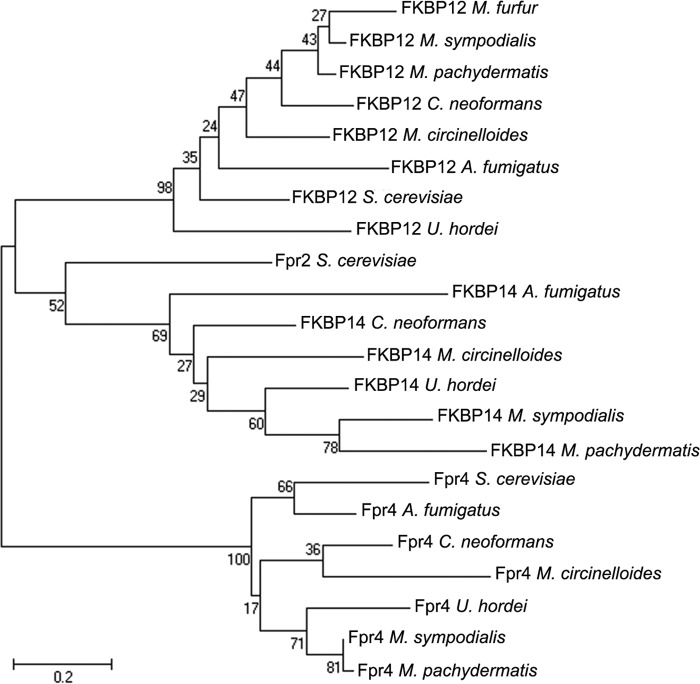
The FKBP proteins identified in the genome of *M. sympodialis* cluster with FKBP orthologs of other representative fungal species. The predicted proteins FKBP12, FKBP14, and Fpr4 of *M. sympodialis*, *M. pachydermatis*, *C. neoformans*, *M. circinelloides*, *A. fumigatus*, *S. cerevisiae*, and *U. hordei* were retrieved from GenBank and used to generate a phylogenetic tree using the maximum likelihood method (see Materials and Methods for details); for *M. furfur*, the predicted FKBP12 protein was also included. The bar shows the number of nucleotide substitutions per site.

Comparative search analysis of the annotated *M. sympodialis* ATCC 42132 genome revealed that four genes share high similarity, with the *S. cerevisiae FPR1* gene encoding FKBP12 with E values of 2e−47 (uncharacterized protein MSY001_0639), 2e−24 (uncharacterized protein MSY001_3308), 1e−22 (uncharacterized protein MSY001_2252), and 1e−22 (peptidyl-prolyl *cis*-*trans*-isomerase [PPIase], similar to *S. cerevisiae* protein Fpr4). Reciprocal BLASTp analysis of the *Saccharomyces* Genome Database (SGD) found FKBP12 to be encoded by MSY001_0639, FKBP14 encoded by MSY001_3308, and Fpr4 encoded by both MSY001_2254 and peptidyl-prolyl *cis*-*trans*-isomerase, with the last being a longer version of Fpr4 that was recently reannotated (30). Further similarity searches among representative fungal genomes and phylogeny analysis confirmed these *M. sympodialis* proteins as being FKBP12, FKBP14, and Fpr4, respectively (Fig. 2). Moreover, using the *S. cerevisiae* gene *FPR1* and its *M. sympodialis* orthologs, the predicted gene encoding FKBP12 of *M. furfur* CBS 14141 was also found in an unpublished PacBio genome assembly and confirmed by phylogenetic clustering (Fig. 2).

Based on its predicted function, the gene encoding FKBP12 in *Malassezia* was named *FKB1* (for FK506-binding protein 1). Targeted mutagenesis of the *M. furfur* and *M. sympodialis FKB1* genes was performed with an *Agrobacterium tumefaciens*-mediated transformation strategy (28). Briefly, gene replacement cassettes comprised of the *NAT* marker and ~1.5-kb regions flanking the *FKB1* gene were fused within the T-DNA region of the binary vector pGI3 by *in vivo* recombination in *S. cerevisiae*. The recombinant plasmids, pGI13 and pGI14, were extracted and inserted into *A. tumefaciens* strain EHA105 for transformation of *M. furfur* and *M. sympodialis*, respectively.

**FIG 3:**
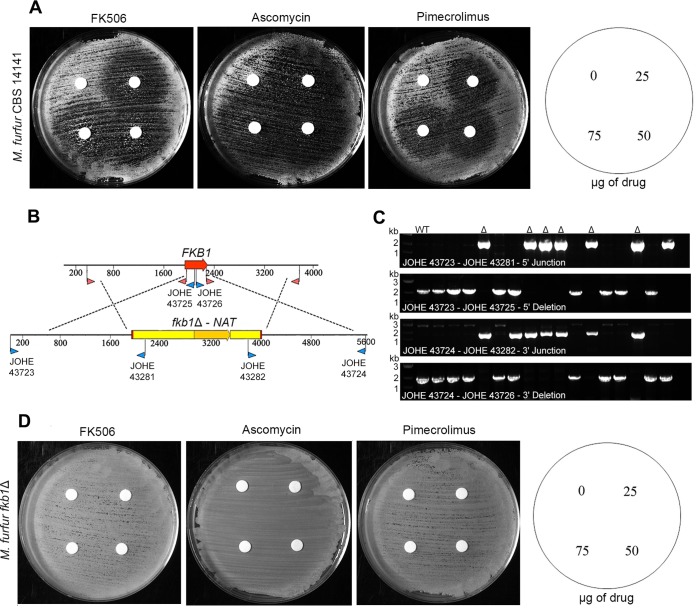
Deletion of the *FKB1* gene of *M. furfur* confers resistance to FK506. (A) Sensitivity of the *M. furfur* strain CBS 14141 to the calcineurin inhibitors FK506, ascomycin, and pimecrolimus. The sensitivity assays in panels A and D were performed at 37°C on mDixon plus 13 mM LiCl with cells exposed to disks containing 0 (vehicle [DMSO]), 25 µg, 50 µg, or 75 µg of FK506 as in Fig. 1. (B) Schematic representation of the targeted gene replacement of the *FKB1* gene of *M. furfur* CBS 14141. Red arrowheads indicate the primers used to amplify the 1.5-kb flanking regions, whereas blue arrowheads indicate screening primers used to identify *fkb1*Δ mutants as shown in panel C. (C) PCR analysis of *M. furfur* WT and nourseothricin-resistant strains for the identification of *fkb1*Δ mutants (indicated by the symbol Δ); primer combinations are indicated in each panel. (D) Full resistance of a representative *M. furfur fkb1*Δ mutant to the calcineurin inhibitors FK506, ascomycin, and pimecrolimus.

For *M. furfur*, the binary vector pGI13 (see Materials and Methods) was designed to replace a region of 312 bp of the *FKB1* gene with the nourseothricin (*NAT*) resistance marker (Fig. 3B). Sixteen NAT-resistant transformants from three independent transformation experiments were selected for further analysis, and six *fkb1*Δ mutants were identified by diagnostic PCR analysis as generating amplicons of the expected size (~2,000 bp) at both the predicted 5′ and 3′ junctions between the gene disruption cassette and the target locus; primers designed within the *FKB1* gene also confirmed targeted gene replacement by the absence of an amplicon for the deletion strains (Fig. 3C). The WT CBS 14141 strain and a representative *fkb1*Δ mutant of *M. furfur* were tested for their phenotype on calcineurin inhibitors employing the conditions developed above (i.e., mDixon plus 13 mM LiCl and 37°C). As expected, the WT displayed clear sensitivity to FK506, ascomycin, and pimecrolimus (Fig. 3A), whereas the *fkb1*Δ mutant was fully resistant to all three drugs (Fig. 3D). All *fkb1*Δ mutants derived from *M. furfur* were confirmed to be resistant to FK506, ascomycin, and pimecrolimus (data not shown).

**FIG 4:**
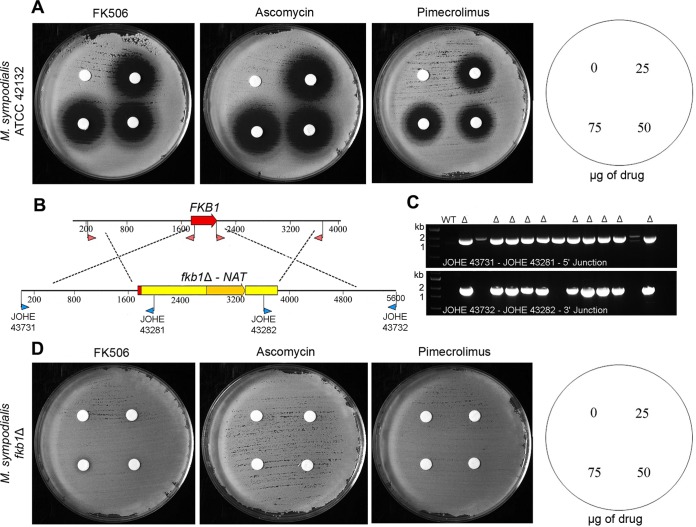
Deletion of the *FKB1* gene of *M. sympodialis* confers resistance to FK506. (A) Sensitivity of the *M. sympodialis* strain ATCC 42132 to the calcineurin inhibitors FK506, ascomycin, and pimecrolimus. The sensitivity assays in panels A and D were performed at 37°C on mDixon plus 15 mM LiCl with cells exposed to disks containing 0 (vehicle), 25 µg, 50 µg, and 75 µg of FK506 as in Fig. 1. (B) Schematic representation of the targeted gene replacement of the *FKB1* gene of *M. sympodialis* ATCC 42132. Red arrowheads indicate the primers used to amplify the 1.5-kb flanking regions, whereas blue arrowheads indicate screening primers used to identify *fkb1*Δ mutants as shown in panel C. (C) PCR analysis of *M. sympodialis* WT and nourseothricin-resistant strains for the identification of *fkb1*Δ mutants (indicated by the symbol Δ); primer combinations are indicated in each panel. (D) Full resistance displayed by a representative *M. sympodialis fkb1*Δ mutant to the calcineurin inhibitors FK506, ascomycin, and pimecrolimus.

Similarly to *M. furfur*, the construct pGI14 (see Materials and Methods) was designed to disrupt the entire *FKB1* open reading frame (ORF) of *M. sympodialis* (replacing 323 bp out of 339 bp) (Fig. 4B). Out of 13 transformants of *M. sympodialis* obtained, 10 were identified by PCR as being *fkb1*Δ mutants (Fig. 4C). Figure 4A and D show the phenotypic differences of the WT strain ATCC 42132 of *M. sympodialis*, which was highly sensitive to FK506, ascomycin, and pimecrolimus using the conditions developed above (i.e., mDixon plus 15 mM LiCl and 37°C), and of the *fkb1*Δ mutant that showed resistance to all three calcineurin inhibitors. All *fkb1*Δ mutants derived from *M. sympodialis* were confirmed to be resistant to FK506, ascomycin, and pimecrolimus (data not shown).

Western blot analysis performed using antiserum raised against the FKBP12 of *S. cerevisiae* detected a band of ~12 kDa for both the WT strains of *M. furfur* and *M. sympodialis*, while no signal was obtained in the corresponding *fkb1*Δ deletion strains, confirming that the loss of FKBP12 confers resistance to the calcineurin inhibitors FK506, pimecrolimus, and ascomycin (Fig. S4).

10.1128/mBio.01752-17.4 FIG S4  FKBP12 is not expressed in the *fkb1*Δ mutants of *M. furfur* and *M. sympodialis*. FKBP12 expression was determined by Western blot analysis of WT strains of *M. furfur* and *M. sympodialis* and of the respective *fkb1*Δ mutants. FKBP12 was detected with antiserum raised against FKBP12 of *S. cerevisiae*. India ink staining of the membrane served as a loading control. Download FIG S4, TIF file, 0.2 MB. Copyright © 2017 Ianiri et al. 2017 Ianiri et al. This content is distributed under the terms of the Creative Commons Attribution 4.0 International license.

### Additive activity of calcineurin inhibitors and fluconazole is dependent on FKBP12 and calcineurin in *M. sympodialis*.

Previous studies reported synergistic effects of calcineurin inhibitors with azoles in *Candida albicans* (31–33), *Cryptococcus neoformans* (34), *Mucorales* (35), and in several species of *Malassezia* (26). For *M. furfur* CBS 14141, the addition of FK506 on paper disks placed on mDixon plus fluconazole (FLC) at several concentrations generated weak growth inhibition halos that, compared to the control (mDixon alone), were more marked at concentrations of 5 µg/ml and 10 µg/ml of FLC, suggesting minimal combined activity of FK506 and FLC (Fig. S5); of note, *M. furfur* CBS 14141 showed, in general, high resistance to FLC with very modest growth inhibition achieved even at 150 µg/ml (data not shown). In contrast, *M. sympodialis* ATCC 42132 was highly sensitive to FLC, and therefore the National Committee for Clinical Laboratory Standards (NCCLS) criteria for antifungal drug activity were applied to accurately determine the MIC at which this drug alone or in combination with calcineurin inhibitors completely inhibited (MIC_100_) the growth of *M. sympodialis* (Table 2). The MICs of FK506, pimecrolimus, and ascomycin were over the highest concentration used in the assay (100 µg/ml), although with a substantial decrease of growth of ~80%, and the MIC of FLC was 1.25 µg/ml. In contrast, for the drug combination the MIC was 0.78 µg/ml for FK506, 1.56 µg/ml for ascomycin, 3.125 µg/ml for pimecrolimus, and 0.625 µg/ml for FLC, with the resulting fractional inhibitory concentration (FIC) index being 0.508, 0.516, and 0.531, respectively, indicating additive activity (Table 2).

10.1128/mBio.01752-17.5 FIG S5  Combined activity of FK506 with FLC against *M. furfur* CBS 14141. The drug sensitivity assay was performed at 37°C on mDixon containing FLC at 0, 1 µg/ml, 5 µg/ml, 10 µg/ml, 25 µg/ml, or 50 µg/ml, and with cells exposed to disks containing 0 (vehicle), 25 µg, 50 µg, or 75 µg of FK506 as indicated in Fig. S1. Download FIG S5, TIF file, 0.6 MB. Copyright © 2017 Ianiri et al. 2017 Ianiri et al. This content is distributed under the terms of the Creative Commons Attribution 4.0 International license.

**TABLE 2:** FK506, ascomycin, and pimecrolimus are additive when combined with fluconazole against *M. sympodialis* in an FKBP12-dependent fashion^a^

*M. sympodialis* strain	MIC (µg/ml) of:							FIC index		
	Agent alone				Agents combined					
	FLC	FK506	Asco	Pime	FLC + FK506	FLC + Asco	FLC + Pime	FLC + FK506	FLC + Asco	FLC + Pime
WT ATCC 42132	1.25	>100	>100	>100	0.625–0.78	0.625–1.56	0.625–3.125	0.508	0.516	0.531
*fkb1*Δ mutant	1.25	>100	>100	>100	1.25–0.195	1.25–0.195	1.25–0.195	1.002	1.002	1.002

^a^ FLC, fluconazole; Asco, ascomycin; Pime, pimecrolimus.

Interestingly, the *fkb1*Δ mutant of *M. sympodialis* showed the same sensitivity to FLC as the WT strain (MIC of 1.25 µg/ml), and as expected, it displayed full resistance to calcineurin inhibitors alone at all of the concentrations tested, with less than 10% of maximum growth inhibition obtained at the highest concentration (100 µg/ml). Drug combinations did not show any synergistic effect against the *fkb1*Δ mutant, with an MIC of 1.25 µg/ml for FLC and 0.195 µg/ml for the calcineurin inhibitors, and a resulting FIC of 1.002, indicating autonomous interaction (Table 2). Paradoxically, the MIC of the calcineurin inhibitors in combination with FLC was higher for the *fkb1*Δ mutant than for the wild type of *M. sympodialis*; however, this was simply due to the toxicity of FLC at a higher dilution in the checkerboard assay (i.e., 2.5 µg/ml) that did not allow the growth of *Malassezia* even when combined with the lowest concentration of calcineurin inhibitors.

**FIG 5:**
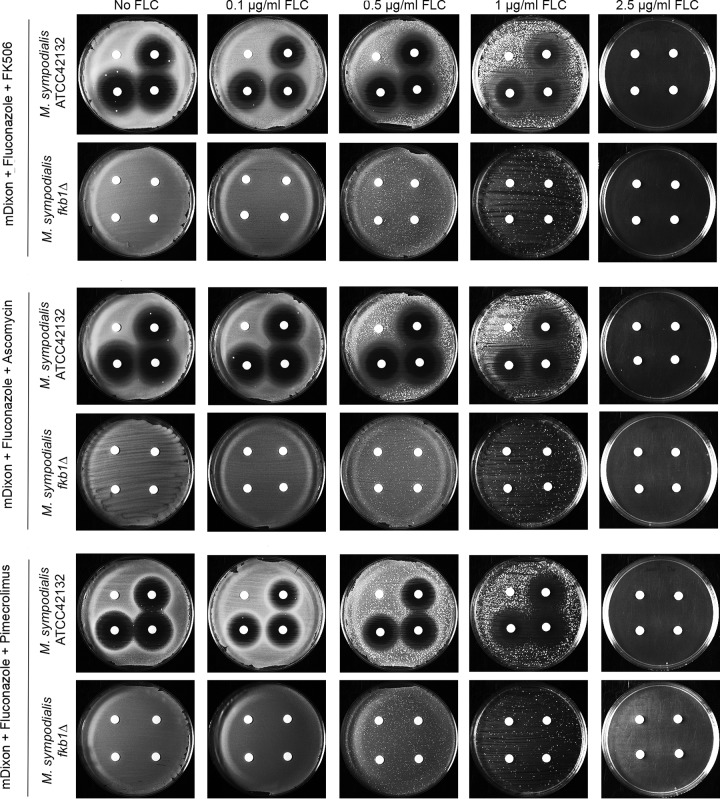
Combined activity of calcineurin inhibitors FK506, ascomycin, and pimecrolimus with FLC against the *M. sympodialis* ATCC 42132 WT strain and the derived *fkb1*Δ mutant. The drug sensitivity assays were performed at 37°C on mDixon containing 0, 0.1 µg/ml, 0.5 µg/ml, 1 µg/ml, or 2.5 µg/ml FLC and with cells exposed to disks containing 0 (vehicle [DMSO]; top left disk), 25 µg (top right disk), 50 µg (bottom right disk), or 75 µg (bottom left disk) of FK506, ascomycin, or pimecrolimus.

Drug sensitivity assessed using disk diffusion assays confirmed the results of the checkerboard assay. In the presence of 25, 50, and 75 µg of FK506, ascomycin, or pimecrolimus, the growth inhibition halos of the *M. sympodialis* WT strain paralleled the concentration increase of FLC, with the strongest inhibition achieved when these calcineurin inhibitors were combined with 1 µg/ml of FLC. In contrast, no growth inhibition halos were observed for the *fkb1*Δ mutant. Finally, 2.5 µg/ml of FLC completely inhibited the growth of both the WT and the *fkb1*Δ mutant (Fig. 5).

### Spontaneous mutant isolates resistant to calcineurin inhibitors have mutations in the *FKB1* gene.

**FIG 6:**
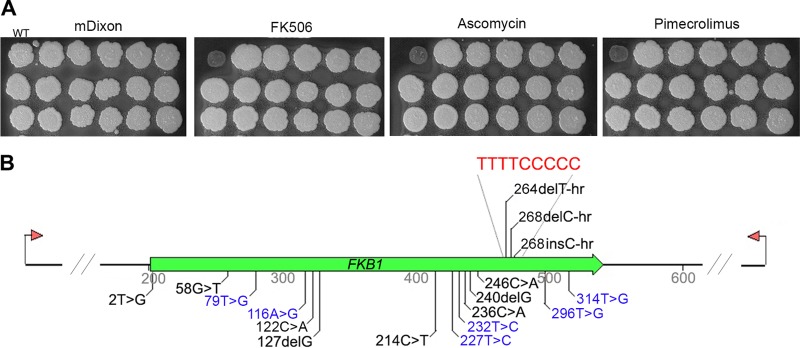
Spontaneous mutations in the *FKB1* gene encoding FKBP12 confer cross resistance to calcineurin inhibitors to *M. sympodialis*. (A) Growth of 17 spontaneous resistant isolates on mDixon plus 15 mM LiCl with no drug (control medium) or supplemented with 25 µg/ml FK506, ascomycin, or pimecrolimus. The WT strain is shown in the top left corner in each panel. (B) Representation of the genetic changes identified in the *FKB1* gene in spontaneous resistant isolates of *M. sympodialis*. The “>” symbol indicates a nucleotide change, with the position of the mutation and the newly acquired nucleotide indicated. del indicates deletion, and ins indicates insertion. Above the schematic representation of the gene, at positions +264 and +268, a 4T-5C contiguous homopolymer repeat is shown in red, and three genetic changes that lie within the homopolymer repeat are shown (indicated as -hr). The mutations that were used for modeling analysis reported in Fig. 7 are shown in blue.

The high sensitivity of *M. sympodialis* to calcineurin inhibitors allowed us to employ a further genetic approach to confirm whether FKBP12 functions as the receptor for these drugs. We isolated a series of spontaneous mutant strains that exhibited resistance to FK506, ascomycin, and pimecrolimus, and examined the *FKB1* gene for mutations. A total of 17 independent strains were isolated, and they all displayed cross resistance to the three drugs (Fig. 6A). The majority of lesions in the sequence of the *FKB1* gene included nucleotide substitutions (12 [70.6%]), four of which were transitions, with two T-to-C substitutions, one A-to-G substitution, and one C-to-T substitution. Eight mutations were transversions, with four T-to-G substitutions, three C-to-A substitutions, and one G-to-C substitution. The remaining mutation events included four single base deletions, and one single C insertion (Fig. 6B). Intriguingly, further analysis of the nucleotide sequence revealed that three of these genetic lesions occurred in a contiguous 4T-5C homopolymer repeat (positions +264 and +268) either by T or C deletion, or by C insertion (Fig. 6B); a deletion in the 5C homopolymer at position +268 was also found for the only FK506-resistant strain isolated from *M. sympodialis* CBS 7222 (data not shown). Overall, the mutations detected resulted in six single amino acid substitutions, one loss of the start codon, nine early stops, and one loss of the stop codon (Fig. S6).

10.1128/mBio.01752-17.6 FIG S6  Multiple alignments of the FKBP12 proteins of the spontaneous resistant isolates of *M. sympodialis* and of the WT FKBP12. Single amino acid substitutions are indicated by gray shading, asterisks indicate stop codons and conserved amino acid residues below the sequence alignment. Download FIG S6, TIF file, 1.1 MB. Copyright © 2017 Ianiri et al. 2017 Ianiri et al. This content is distributed under the terms of the Creative Commons Attribution 4.0 International license.

**FIG 7:**
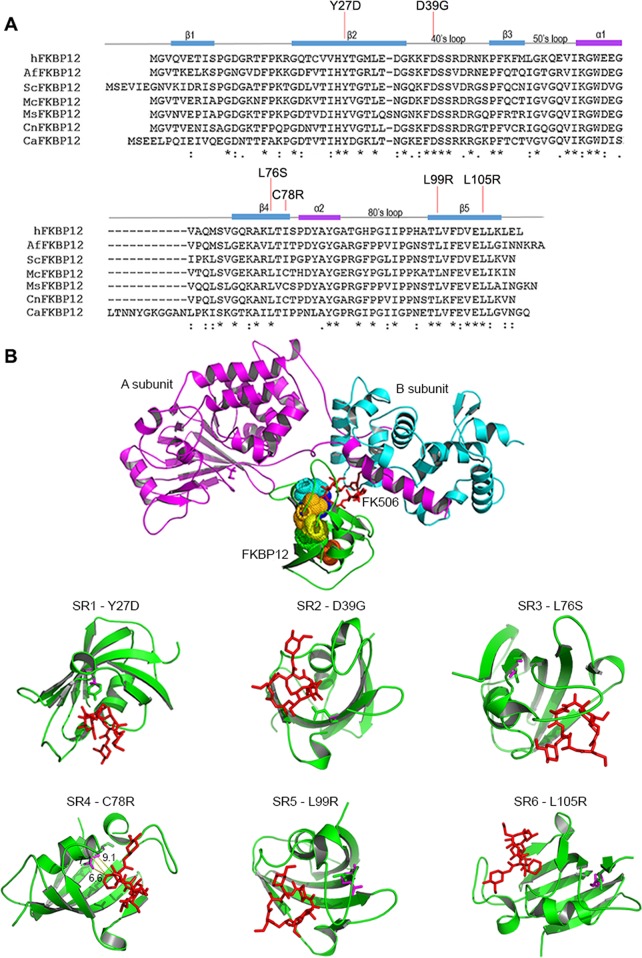
Structure modeling of mutated FKBP12 in spontaneous isolates resistant to calcineurin inhibitors. (A) Sequence alignment of the human (h), *Aspergillus fumigatus* (Af), *S. cerevisiae* (Sc), *Mucor circinelloides* (Mc), *M. sympodialis* (Ms), *C. neoformans* (Cn), and *C. albicans* (Ca) FKBP12 proteins. Secondary structural elements are shown above the sequence alignment based on the article by Tonthat et al. (37). Conserved amino acid residues (asterisks), conservation between groups of strongly similar properties (scoring > 0.5 in the Gonnet PAM 250 matrix) (colons), and conservation between groups of weakly similar properties (scoring ≤ 0.5 in the Gonnet PAM 250 matrix) (periods) are indicated below the sequence alignment. (B) Homology modeling of calcineurin complex (Cna1-Cnb1-FK506-FKBP12) of *M. sympodialis* and effect of amino acid substitutions on FK506 susceptibility. Cna1 (violet), Cnb1 (blue), FKBP12 (green). Bubbles on FKBP12 indicate the amino acids that were altered by mutations. FK506 is shown in red.

We further focused on the spontaneous resistant isolates having lesions in the *FKB1* gene that resulted in nonsynonymous mutations and examined how they acquired resistance to calcineurin inhibitors through modeling of the calcineurin-inhibitor complexes. Multiple comparison of the human FKBP12 with that of the model yeast *S. cerevisiae* and the human-pathogenic fungi *Aspergillus fumigatus*, *Mucor circinelloides*, *C.  neoformans*, and *C. albicans*, allowed prediction of the characteristic five β-sheets, central α-helixes, and 40s, 50s, and 80s loops of *M. sympodialis* FKBP12. Mutations were found in the β2-sheet (Y27D), in the 40s loop (D39G), in the β4-sheet (L76S and C78R), and in the β5-sheet (L99R, L105R) (Fig. 7A).

Next, a projected *M. sympodialis* model of the calcineurin complex inhibited by FKBP12-FK506 was built using SWISS-MODEL (36) based on homology with the amino acid sequences of the calcineurin A and B subunits and with FKBP12 constructed based on a crystal structure of the *Coccidioides immitis* FK506-inhibited calcineurin complex (Seattle Structural Genomics Center for Infectious Disease [SSGCID] et al., unpublished; SWISS-MODEL template library [SMTL] identifier [ID] or accession number 5b8i.1) (Fig. 7B and Movie S1). Note that two Cna1 and Cnb1 predictions were available in GenBank, as based on previous and updated genome annotations (12, 30); for both proteins, the most recent versions were used (Cna1, accession number SHO80045; Cnb1, accession number SHO76711). Each amino acid substitution was evaluated in the *M. sympodialis* FKBP12-FK506 complex built with the *A. fumigatus* FKBP12-FK506 complex (37) (Fig. 7B). In the SR1 mutant, the substitution of a hydrophobic tyrosine at the 27th residue with a negatively charged aspartic acid likely resulted in a repulsive effect between FKBP12 and FK506. In the SR2 mutant, the substitution of a charged aspartic acid with a small glycine at the 39th residue within the 40s loop may have changed the interaction pattern between FKBP12 and FK506. Based on the model built, the lysine at the 76th residue is less likely to interact directly with FK506; however, in the SR3 mutant, the substitution of lysine by serine might have resulted in a conformational change of FKBP12, thus perturbing the interaction between FKBP12 and FK506. In the SR4 mutant, the distances from the 78th cysteine residue in the WT or from the 78th arginine residue in the mutant and the FK506 molecule are relatively long (9.1 Å and 7.1 or 6.6 Å, respectively), suggesting that steric clash resulting from the amino acid substitution may not have occurred. Instead, the Cys-to-Arg substitution at the 78th residue may cause a conformational change in FKBP12, hence affecting the binding affinity between FKBP12 and FK506. In the SR5 and SR6 mutants, substitution of lysine to arginine at the 99th and 105th residues, respectively, may cause a conformational change in FKBP12 that perturbs the interaction between FKBP12 and FK506. Because both lysine and arginine are positively charged, but distant from the hydrophobic drug binding pocket, they might alter the conformation of the pocket rather than its hydrophobicity and thereby confer drug resistance. Overall, the predicted models indicated that the amino acid alterations in the FKBP12 protein are less likely to affect the interaction between calcineurin AB subunits and the FKBP12-FK506 complex, but rather they affect the interactions between FKBP12 and FK506 or the stability of FKBP12.

10.1128/mBio.01752-17.9 MOVIE S1  Homology modeling of FKBP12-FK506-inhibited calcineurin of *M. sympodialis*. Download MOVIE S1, MOV file, 6.7 MB. Copyright © 2017 Ianiri et al. 2017 Ianiri et al. This content is distributed under the terms of the Creative Commons Attribution 4.0 International license.

### An *M. sympodialis msh2*Δ hypermutator exhibits an elevated rate of evolution of resistance to FK506 compared to the wild type.

To investigate the effects of a defective mismatch repair system (MMR) in the adaptation of *M. sympodialis* exposed to the calcineurin inhibitor FK506, a mutation in the MMR gene *MSH2* was generated. The *M. sympodialis MSH2* gene was identified in the genome assembly using the yeast *MSH2* as a query; note that all of the orthologs of the yeast MMR system (*MSH2*, *MSH3*, *MSH6*, and *MLH1*) are present in the *M. sympodialis* genome. Using the same procedure described for the *FKB1* genes, an *msh2*Δ::*NAT* gene replacement allele was generated and successfully introduced using *A. tumefaciens* to generate an *M. sympodialis msh2*Δ mutant (Fig. S7).

10.1128/mBio.01752-17.7 FIG S7  (A) Schematic representation of the targeted gene replacement of the *MSH2* gene of *M. sympodialis* ATCC 42132. Blue indicates the coding region of MSH2, while grey indicates the intron. Red arrowheads indicate the primers used to amplify the 1.5-kb flanking regions, whereas blue arrowheads indicate screening primers used to identify *msh2*Δ mutations as shown in the PCR in panel B. (B) PCR analysis of *M. sympodialis* WT and nourseothricin-resistant strains for the identification of *msh2*Δ mutants (indicated by the Δ symbol); primer combinations are indicated in each panel. Download FIG S7, TIF file, 0.2 MB. Copyright © 2017 Ianiri et al. 2017 Ianiri et al. This content is distributed under the terms of the Creative Commons Attribution 4.0 International license.

**FIG 8:**
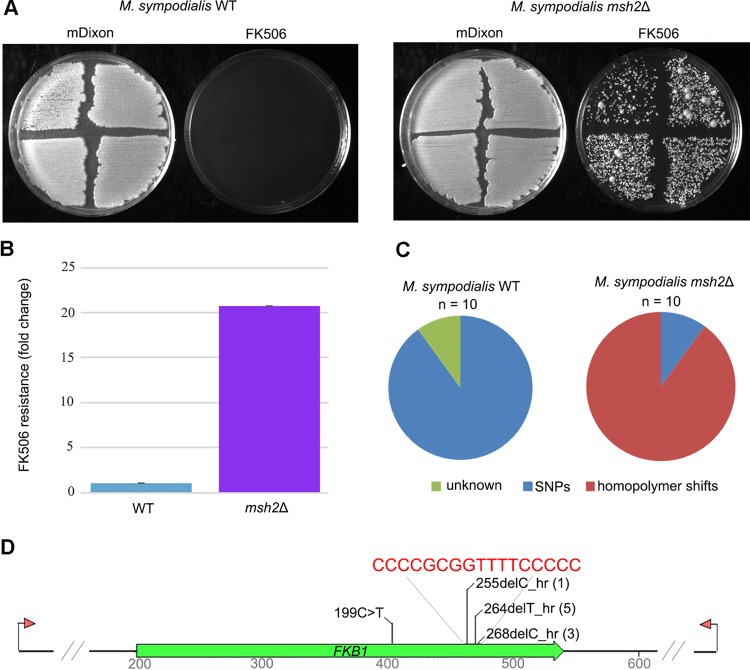
An *msh2*Δ hypermutator of *M. sympodialis* displays increased emergence of resistance to FK506 compared to the WT strain. (A) Qualitative phenotypic analysis of the *M. sympodialis* WT strain and its derived *msh2*Δ mutant on mDixon plus 15 mM LiCl with 25 µg/ml FK506 or without FK506. (B and C) FK506 resistance rate expressed as fold change increase resulting from a classical fluctuation assay performed using the *M. sympodialis* WT strain and its derived *msh2*Δ mutant (B), and genetic lesions identified in 10 independent FK506-resistant isolates derived from both background strains (C). SNPs, single nucleotide polymorphisms. (D) Representation of the mutations identified in the *FKB1* gene in FK506-resistant isolates derived from the *msh2*Δ mutant background. A region of the *FKB1* gene indicated in red contains three homopolymer repeats (hr), 4C at position +255, and a contiguous 4T-5C homopolymer repeat at positions +264 and +268, in which the mutations were found. The number of independent mutational events are indicated in parentheses after the mutation.

As a first step, a qualitative drug resistance assay was performed by swabbing four independent colonies of the *M. sympodialis* WT strain and of the *msh2*Δ mutant on control media (mDixon plus 15 mM LiCl) and FK506-containing media (mDixon plus 15 mM LiCl and 25 µg/ml FK506). As expected, at a temperature of 37°C, while both strains grew at similar rates on control media, a number of FK506-resistant colonies appeared only in the *msh2*Δ mutant background, with no FK506-resistant colony observed for the WT strain (Fig. 8A). As a further step, classical fluctuation assays were performed using 10 independent replicates of both strains, revealing an increase of more than 20-fold in the emergence of FK506 resistance for the *msh2*Δ mutant compared to the *M. sympodialis* WT type strain (Fig. 8B). Genetic analysis of the *FKB1* locus of 10 independent FK506-resistant isolates derived from both strain backgrounds detected 9 single nucleotide polymorphisms (SNPs) and an uncharacterized mutation for the WT, whereas only 1 SNP (199C>T) and 9 single base deletions in homopolymer repeats (i.e., homopolymer shifts) were detected in the *msh2*Δ mutant (Fig. 8C). In strains derived from the *msh2*Δ mutant, sequence analysis revealed one mutation of a 4C run at position +255 and five and three mutations of the contiguous 4T-5C runs at positions +264 and +268, respectively (Fig. 8C). In all of these FK506-resistant isolates, the homopolymer shift led to a predicted premature stop codon in the gene encoding the FKBP12 protein.

## DISCUSSION

In the present study, the mode of action of calcineurin inhibitors in *M. furfur* and *M. sympodialis* was investigated. These two species are model species of two main subgroups within the *Malassezia* genus and can be considered representative of the fungemia-causing and commensal groups, respectively (5, 6). These two species were also selected because gene disruption systems have been established (28). A third species, *M. pachydermatis*, is also amenable to transformation (38), but the work on FKBP12 was initiated before this information became available. As a first step, it was crucial to identify the conditions under which calcineurin plays an essential role for the cell. Initial drug sensitivity assays for *M. furfur* and *M. sympodialis* were performed under conditions reported in the literature as critical for FK506 toxicity, i.e., at 37°C as reported for *C. neoformans* (39), alone or in the presence of salts (LiCl), the latter reported for *S. cerevisiae* (29). We found that in all cases a temperature of 37°C was required for FK506 toxicity against both *M. furfur* and *M. sympodialis*, while the presence of LiCl led to different phenotypic responses among the strains tested: it was essential for clear toxicity of FK506 against *M. furfur* CBS 14141 (Fig. 1A) and *M. sympodialis* CBS 7222 (Fig. S3), and it increased the toxicity of FK506 against *M. sympodialis* ATCC 42132 and ATCC 44340 strains (Fig. 1B and Fig. S1 in the supplemental material). Interestingly, under these optimal conditions, *M. furfur* and *M. sympodialis* displayed different responses to the toxicity of FK506, with *M. furfur* showing inhibition halos with large diameters but with residual growth, and *M. sympodialis* showing clear halos rather than turbidity, but with reduced diameters (Fig. 1). This may suggest that FK506 displays a fungistatic effect against *M. furfur* and rather a fungicidal effect against *M. sympodialis*. In spite of the differences between the two species studied, the data obtained clearly indicate that calcineurin in *Malassezia* is required for growth at elevated temperatures, a function shared with the human pathogen *C. neoformans* (39). This could indicate that calcineurin in *Malassezia* plays a role in pathogenesis (i.e., fungemia), as the ability to grow at human body temperature is a necessary feature for virulence in pathogenic fungi. Moreover, the toxicity of calcineurin inhibitors combined with LiCl (Fig. 1, S1, S3, 2A, and 3A) and NaCl (data not shown) suggests a further role of calcineurin in cation homeostasis and plasma membrane functionality, a function shared with *S. cerevisiae* (29), although its importance in *Malassezia* might vary between species and even between strains of the same species.

As a next step, we demonstrated that the antifungal activity of calcineurin inhibitors occurs via the binding protein FKBP12. Using an *A. tumefaciens*-mediated transformation approach that we recently developed (28), a high frequency of homologous recombination was achieved to successfully replace the gene encoding FKBP12 (named *FKB1*) in both *M. furfur* and *M. sympodialis*. As expected, the *fkb1*Δ mutants displayed full resistance to FK506, ascomycin, and pimecrolimus (Fig. 3 and 4), indicating the highly conserved role of FKBP12 across eukaryotic organisms (23, 25).

We found that the calcineurin inhibitors FK506, ascomycin, and pimecrolimus act additively with fluconazole (FLC) versus *M. sympodialis* ATCC 42312, and that this mechanism is mediated by FKBP12 and thus mediated by calcineurin. This finding was supported by the full resistance of the *M. sympodialis fkb1*Δ mutant to calcineurin inhibitors and by the increased resistance to FLC combined with calcineurin inhibitors compared to the WT (Table 2 and Fig. 5). Based on the results described above, it is likely that the mechanism observed is due to the essential role of calcineurin during membrane stress, in this case caused by FLC, which in addition could enhance the uptake of calcineurin inhibitors by increasing their penetration in the cell and/or by compromising the multidrug resistance (MDR) pump function. Our hypothesis is also corroborated by previous findings in *C. albicans*, in which calcineurin function becomes essential when plasma membrane integrity is compromised, resulting in a synergistic effect of FK506 combined with FLC that is dependent on calcineurin (31). A synergistic antifungal effect of FLC with calcineurin inhibitors has been reported for the human pathogen *C. neoformans*, but in this case, it is FKBP12 and calcineurin independent and might involve multidrug resistance pumps (34). A previous study reported synergistic activity of ketoconazole and itraconazole combined with FK506 against several species of *Malassezia*, including *M. furfur* and *M. sympodialis* (26). However, compared to this study, we observed an additive activity for *M. sympodialis* (i.e., higher FIC index) (Table 2) and no relevant effect of FK506 combined with FLC against *M. furfur* (Fig. S5). The different results obtained by Sugita and colleagues (26) might be due to different experimental conditions used (medium and temperature), different strain backgrounds tested (strain names are not available in reference 26), or different azoles, the last being supported by previous findings in pathogenic zygomycetes in which combinations of posaconazole and itraconazole with FK506 increase fungicidal activity by inducing cell apoptosis, whereas FK506 combined with FLC had no relevant effect (40).

From a clinical viewpoint, although the topical calcineurin inhibitors tacrolimus (FK506, Protopic) and pimecrolimus (Elidel) are available in the clinic as alternative to corticosteroids to treat inflammatory skin conditions, our microbiological drug assays revealed that a practical limitation to their use is that their maximum toxicity is exerted at 37°C, while human skin temperature is on average 32°C to 34°C (41). Nonetheless, calcineurin inhibitors still showed antifungal activity at temperatures lower than 37°C against *M. sympodialis* (Fig. S2), which is most commonly associated with clinical skin disorders, and their immunosuppressive activity is also required for inflammatory conditions (15, 19). Moreover, our study and other studies revealed that calcineurin inhibitors act additively and/or synergistically with fluconazole, itraconazole, and ketoconazole (26), and hence to enhance antifungal effect, they could be prescribed in combination with these antifungal azoles, contributing also at reducing the emergence of azole-resistant strains. Finally, it may be the case that conditions on the skin might render fungal calcineurin important for growth in response to other stresses present in the skin microenvironment.

By taking advantage of the strong fungicidal effect of calcineurin inhibitors against *M. sympodialis*, strains that acquired spontaneous and cross resistance to FK506, ascomycin, and pimecrolimus were isolated (Fig. 6A); this genetic approach could not be performed in *M. furfur* due to the fungistatic effect of calcineurin inhibitors that did not allow the clear identification of resistant strains. In *M. sympodialis*, the spontaneous resistant strains had mutations in the *FKB1* gene (Fig. 6B) that resulted in the production of altered FKBP12 proteins due to single amino acid substitutions, loss of the start or stop codons, or premature terminator codon (Fig. S6). Multiple alignments of the human and fungal FKBP12 proteins (37) allowed the prediction of the classical β-sheets, α-helixes, and loop regions of the *M. sympodialis* FKBP12 and the regions impacted by nonsynonymous amino acid substitutions (Fig. 7A).

Structural homology modeling of the *M. sympodialis* FKBP12-FK506 calcineurin complex and analysis of the FK506-resistant isolates, combined with FKBP12 protein analysis, revealed how these changes within the FKBP12 sequence affected the overall structure of the calcineurin inhibitory complex. All changes impacted conserved amino acid residues, with the only exception being C78R (Fig. 7A), and all resulted in loss of function with respect to drug action. Substitutions of the tyrosine at position 27 to aspartic acid (Y27D) and of the aspartic acid at position 39 to glycine (D39G) are mutations known to prevent the binding between FK506 and FKBP12, as confirmed also by modeling analysis (Fig. 7B). Michnick and colleagues identified the tyrosine at the 26th residue of human FKBP12 (corresponding to tyrosine 27 in *M. sympodialis*) as part of the aromatic cluster that forms a hydrophobic core of FKBP12 required for binding FK506 (42). Moreover, FKBP12 is characterized by three loops (40s, 50s, and 80s) that surround a pocket that binds FK506 and rapamycin (37, 42, 43). Mutation of the aspartic acid at position 37 in the 40s loop of the human FKBP12 (corresponding to aspartic acid at position 39 in *M. sympodialis* FKBP12) was found to reduce peptidyl-prolyl isomerase activity and reduced FK506 binding compared to the control due to disruption of the electrostatic interactions formed by the D37 carboxylate oxygen and specific residues on FK506 (44, 45). Corresponding mutations of the conserved tyrosine 26 and aspartic acid 37 were also identified in FK506-resistant isolates derived from the zygomycete fungus *Mucor circinelloides* (46). A number of other mutations within conserved structural regions of FKBP12 have been shown to confer FK506 resistance in fungi, including the pathogenic *Mucorales M. circinelloides* (46–48), *C. neoformans* (39), and *A. fumigatus* (37). These findings and our findings support the model in which the nonsynonymous mutations of *M. sympodialis* FKBP12 conferred FK506 resistance by affecting the interactions between FKBP12 and FK506 and/or the stability of FKBP12.

Resistance to calcineurin inhibitors in fungi is also acquired through mutations in the FK506-FKBP12 complex binding domain of the calcineurin subunit A or B (49–52), and their identification is facilitated by the sensitivity displayed by the strains under examination to rapamycin, another immunosuppressive drug that forms a toxic complex with FKBP12 and targets calcineurin and mTOR (53, 54). Unfortunately, *M. furfur* and *M. sympodialis* were not sensitive to rapamycin, and this likely hindered the isolation and identification of any FK506-resistant strain with genetic lesions in the *CNA1* (calcineurin subunit A) and *CNB1* (calcineurin subunit B) genes. In our study, only one FK506-resistant isolate having no mutation in the *FKB1* gene was isolated during fluctuation assays (Fig. 8C), but further analysis revealed that neither calcineurin subunit-encoding gene had mutations. This situation in *M. circinelloides* revealed a unique RNA inhibition (RNAi)-dependent epimutation mechanism that confers transient FK506 resistance (46), but in *Malassezia* this phenomenon can be excluded due to the lack of RNAi components (5); thus, FK506 resistance in this uncharacterized isolate may be due to mutations affecting genes encoding components of multidrug efflux pumps or other mechanisms.

Due to the isolation of calcineurin inhibitor-resistant isolates of *M. sympodialis* with genetic mutations in homopolymer repeats (Fig. 6B), we hypothesized that we could generate an *M. sympodialis* hypermutator strain defective in the replication of these specific sequences and score the expected increase in the mutational rate using the drug FK506. Based on studies of yeast and of the human pathogens *C. neoformans* and *Cryptococcus gattii*, Msh2 is part of the DNA mismatch repair (MMR) system that plays an important role in maintaining normal mutation rates. Either *msh2*Δ null mutants or naturally occurring *msh2* mutants are characterized by a mutator phenotype with an elevated mutation rate in homopolymer repeats (55–58). In agreement with these studies, an *M. sympodialis msh2*Δ mutant showed a hypermutator phenotype with an increase of more than 20-fold in FK506 resistance compared to the WT (Fig. 8A and B); 90% of the mutations that conferred resistance to FK506 consisted of homopolymer shifts within the *FKB1* gene, with 10% in the 4C homopolymer at position +255, 50% in the 4T homopolymer at position +264, and 30% in the 5C homopolymer at position +268 (Fig. 8C and D). Conversely, FK506 resistance in the WT was acquired through a wide range of genetic lesions (Fig. 6A and B, and 8C), including all except G-to-A transitions, and four different mutations that impacted the 4T-5C homopolymer repeat (+264 to +268). Our results differ slightly from recent findings in *C. neoformans*, in which transitions in the *URA5* gene were all T-to-C substitutions and homopolymer shifts in the *FUR1* gene were all deletion of the A6 homopolymer tract (57). Although it seems that the mechanisms that control the type of mutations in these basidiomycete yeasts are different, it is likely that these conflicting results are also due to the intrinsic features of the markers used: the *FKB1* gene used in our study is 339 bp, has no introns, and the longest homopolymer run is 5C (at +268). Conversely, the genetic markers (*URA5* and *FUR1*) used in the study of Boyce and colleagues (57) are longer than 1 kb and have multiple introns, and in the case of *FUR1*, long homopolymer repeats (C7, A6, and T14) are also present.

The rationale of studying the mutator phenotype is due to the natural occurrence of strains having spontaneous defects within the *MSH2* gene in fungal populations of the human pathogens *C. neoformans* (57), *C. gattii* (58), and *Candida glabrata* (59); these strains are likely the result of environmental or pharmacological selective pressure that requires a high adaptation capability for microevolution. Clinical hypermutator strains were isolated at a significant frequency from infected hosts, and both *in vitro* and *in vivo*, they were able to rapidly acquire resistance to a different class of antifungal chemicals and quickly adapt to host conditions. These findings have clinical implications for antifungal prophylaxis and antifungal protocols. In the present study, we have generated the first known hypermutator strain in the *Malassezia* genus, and at this time, a large-scale screening of clinical isolates for hypermutator phenotypes is in progress.

In conclusion, we demonstrated a conserved mechanism for calcineurin inhibitors that exert antifungal activity against *M. furfur* and *M. sympodialis* by forming a toxic complex with the immunophilin FKBP12 that targets calcineurin and blocks its signaling activity. Overall, our study further reinforces previous research aimed at development of novel calcineurin inhibitors with lower immunosuppressant activity that in novel formulations can increase permeability of these drugs (i.e., nanoparticles) and can be used in combination with existing antifungals to treat a broad range of fungal infections, including skin disorders caused by *Malassezia* and other fungi.

## MATERIALS AND METHODS

### Fungal strains.

For antifungal drug assays, the *Malassezia furfur* CBS 14141 and CBS 7982 strains and the *Malassezia sympodialis* ATCC 42132, ATCC 44340, and CBS 7222 strains were used. Unless stated otherwise, these strains were grown on modified Dixon’s medium (mDixon), which is the medium routinely used for culturing *Malassezia* species (5). All fungal strains used and generated in the present study are listed in Table 1.

### Antifungal drug assays.

FK506 (Sigma) was dissolved in dimethyl sulfoxide (DMSO) and added to the media at the desired concentration. For the antifungal drug assays, an aliquot (300 µl) of overnight (ON) cultures of *M. furfur* and *M. sympodialis* strains was either spread or swabbed onto mPDA or mDixon agar and allowed to dry; subsequently, an appropriate volume (from 5 to 15 µl) of solvent containing 0, 1, 5, 10, 25, 50, or 75 µg of FK506 was spotted directly on a blank paper disk (diameter, 6 mm; Fisher Scientific) placed on the agar surface. The plates were incubated at 24°C, 30°C, and 37°C for 4 days. Subsequently, the same assay was performed on mPDA and mDixon agar supplemented with the following chemicals at the indicated concentrations: lithium chloride (LiCl) at 5 mM, 10 mM, 12 mM, 13 mM, 15 mM, 20 mM, 30 mM, 50 mM, 75 mM, or 100 mM; sodium chloride (NaCl) at 0.5 M, 1 M, or 1.5 M; fluconazole (FLC) at 0.1 μg/ml, 0.5 μg/ml, 1 μg/ml, 2.5 μg/ml, 5 μg/ml, 10 μg/ml, 25 μg/ml, or 50 μg/ml. Finally, using the conditions in which FK506 showed antifungal effects (i.e., mDixon plus 13 mM or 15 mM LiCl), 25 μg, 50 μg, and 75 μg of ascomycin (MedChem Express) and pimecrolimus (Cayman Chemical) dissolved in DMSO were also tested.

The interaction of calcineurin inhibitors FK506, ascomycin, and pimecrolimus with FLC was assessed with a checkerboard titration assay, according to the National Committee for Clinical Laboratory Standards (NCCLS), and a similar experiment performed for several *Malassezia* species (26, 31). Drugs were diluted in 200 µl of liquid mDixon and arranged in 96-well plates to make a dilution series of 70 drug combinations, plus additional rows containing each agent alone for MIC determination, and for the growth control (drug free). The final drugs and drug concentrations tested were FLC from 10 µg/ml to 0.156 µg/ml and calcineurin inhibitors from 100 µg/ml to 0.195 µg/ml. The yeast inocula were prepared from ON cultures and added to each well to an optical density of 600 nm (OD_600_) of 0.2, corresponding to ~2 × 10^8^ CFU/ml; microtiter plates were incubated at 37°C without shaking. The OD_600_ values were obtained following 7 days of incubation using a microplate reader (Sunrise Tecan), with 10 s of shaking before reading. The MIC of both drugs, alone or in combination, was defined as the lowest drug concentration in a well which produced an inhibition or decrease in absorbance of >95% compared with that of the growth control well. Drug interactions were classified as synergistic or indifferent on the basis of the fractional inhibitory concentration (FIC) index. The FIC index is the sum of the FICs for each of the drugs, which in turn is defined as the MIC of each drug when used in combination divided by the MIC of the drug when used alone. The interaction was defined as synergistic if the FIC index was <0.5, additive if the index was >0.5 and <1.0, autonomous if the FIC index was between 1.0 and 2.0, and antagonist if the FIC index was >2.0.

### Targeted gene replacement in *M. sympodialis* and *M. furfur* through *Agrobacterium tumefaciens*-mediated transformation.

The *M. sympodialis FKB1* gene encoding FKBP12 was identified in the newly released genome assembly (30) though BLASTp analysis using the FKBP12 protein sequence of *Saccharomyces cerevisiae* (encoded by the *FPR1* gene), which was retrieved from the *Saccharomyces* Genome Database (SGD). Both *M. sympodialis* and *S. cerevisiae* FKBP12 were used as queries for tBLASTn analysis to identify the predicted *FKB1* gene in the unpublished PacBio genome assembly of *M. furfur* CBS 14141. Sequences were subjected to reciprocal BLAST analyses on GenBank and SGD. To unequivocally assign predicted functions based on sequence similarity, the gene encoding FKBP12 of *M. furfur*, and the genes predicted to encode FKBP12, FKBP14, and Fpr4 in *M. sympodialis*, *Malassezia pachydermatis*, *S. cerevisiae*, *Cryptococcus neoformans*, *Aspergillus fumigatus*, *Ustilago hordei*, and *Mucor circinelloides* were retrieved from GenBank, aligned using the MUSCLE software program, and uploaded into MEGA7 (60) for phylogenetic analysis using the maximum likelihood method (WAG model, five discrete gamma categories) and 100 bootstrap replications. Similarly, the DNA mismatch repair binding protein Msh2 was identified in the *M. sympodialis* genome assembly following BLASTp analysis using *S. cerevisiae* Msh2 as a query and confirmed by reciprocal BLAST analysis.

Binary vectors for *A. tumefaciens*-mediated targeted gene replacement of the *FKB1* gene of *M. sympodialis* and *M. furfur* and of *MSH2* of *M. sympodialis* were assembled in *S. cerevisiae* using the binary vector pGI3 as previously reported (28, 61). The *NAT* cassette (p*ACT1-NAT-*t*ACT1*) was amplified from plasmid pAIM1 using primers ALID2078 and ALID2081. Flanking regions of ~1.5 kb upstream and downstream of the target *FKB1* genes were amplified from genomic DNA of *M. furfur* CBS 14141 using primer pairs JOHE43269-JOHE43270 and JOHE43271-JOHE43272, respectively, and from genomic DNA of *M. sympodialis* ATCC 42132 using primer pairs JOHE43727-JOHE43728, and JOHE43729-JOHE43730, respectively. Similarly, the ~1.5-kb upstream and downstream regions flanking the *MSH2* gene of *M. sympodialis* were amplified using primer pairs JOHE44256-JOHE44257 and JOHE44258-JOHE44259, respectively. The PCR products and the doubly digested (KpnI and BamHI) binary vector pGI3 were transformed into *S. cerevisiae* using lithium acetate and polyethylene glycol 3750 (PEG 3750) as previously reported (28).

To assess correct recombination of the newly generated plasmids, single colonies of *S. cerevisiae* transformants were screened by PCR using primers specific for the *NAT* marker (GI154-GI155) in combination with primers designed on the backbone plasmid pGI3 (GI152-GI153). Positive clones of *S. cerevisiae* were grown ON in yeast extract-peptone-dextrose (YPD) and subjected to phenol-chloroform-isoamyl alcohol (25:24:1) plasmid extraction using the protocol reported by Hoffman (62). The plasmid DNA obtained was then introduced into the *A. tumefaciens* EHA105 strain by electroporation, and the transformants were selected on Luria-Bertani (LB) plus 50 µg/ml kanamycin.

*A. tumefaciens*-mediated transformation was performed using the optimized protocol that we previously developed with slight modifications (28). Briefly, *M. furfur* and *M. sympodialis* were grown ON in mDixon at 30°C and then adjusted to an OD_600_ of ~1; the engineered *A. tumefaciens* strains were grown ON in Luria-Bertani plus 50 µg/ml of kanamycin at 30°C and then diluted to an OD_600_ of ~0.8 to 1. Four mixes of these fungal and bacterial cell suspensions at the indicated OD values or diluted further to 1/10 were spotted directly on nylon membranes placed on modified induction medium (mIM) agar (28). Coincubation cultures were kept at room temperature for 3 and 6 days without Parafilm prior to transferring the dual cultures to mDixon supplemented with nourseothricin (100 µg/ml) to select for fungal transformants and with cefotaxime (200 µg/ml) to inhibit *Agrobacterium* growth.

Transformants were purified to single colonies and subjected to phenol-chloroform-isoamyl alcohol (25:24:1) DNA extraction (62), and the correct replacement of the target locus was assessed by PCR. *fkb1*Δ mutants of *M. furfur* were identified with primer JOHE43273 or JOHE43274 in combination with specific primers for the *NAT* gene (JOHE43281 and JOHE43282, respectively) and with primers JOHE43275-JOHE43276 that are targeting regions within the wild-type (WT) *FKB1* gene. Similarly, homologous recombination events in the *fkb1*Δ mutants of *M. sympodialis* were identified with primer pairs JOHE43731-JOHE43281 and JOHE43732-JOHE43282, while the absence of the WT copy of the *FKB1* gene was assessed with primer pair JOHE43733-JOHE43734. For the identification of *msh2*Δ mutants, primer pairs JOHE44260-JOHE43281, JOHE44261-JOHE43282, and JOHE44262-JOHE44263 were used. In all cases, 33 cycles of PCR amplification was performed, with 1 cycle consisting of denaturation at 94°C for 30 s, annealing at 55°C for 30 s, and extension at 72°C of 1 min per kb, with an initial denaturation step at 94°C for 2 min and a final extension step at 72°C for 5 min. PCR analyses were performed using ExTaq (Takara) according to the manufacturer’s instructions; when this polymerase did not work, LA Taq (Takara) with buffer I and buffer II was used. Primer sequences are listed in Table S1 in the supplemental material.

10.1128/mBio.01752-17.8 TABLE S1  Primer sequences (5′-3′) used in the present study. Download TABLE S1, XLSX file, 0.01 MB. Copyright © 2017 Ianiri et al. 2017 Ianiri et al. This content is distributed under the terms of the Creative Commons Attribution 4.0 International license.

### Molecular characterization of spontaneously drug-resistant isolates of *M. sympodialis*.

Spontaneous FK506-, ascomycin-, and pimecrolimus-resistant strains of *M. sympodialis* ATCC 42132 were independently isolated following two different procedures. (i) Spontaneous resistant strains were isolated as single colonies that appeared within the inhibition halos caused by the antifungal activity of the drugs during the disk diffusion assays that aimed at the optimization of the conditions in which calcineurin is essential. (ii) Spontaneous resistant strains were isolated from mDixon agar containing 13 mM LiCl and 25 μg/ml of each drug inoculated with an aliquot (300 µl) of independent ON culture (one culture per agar plate). Colonies were purified and subjected to DNA extraction as reported above. Genomic DNA was subjected to PCR analysis using primer pair JOHE43735-JOHE43736 to amplify a region of 1 100 bp that included the *FKB1* open reading frame (ORF) and ~300-bp sequence upstream and downstream of the gene. Thirty-three cycles of PCR amplification was performed, with 1 cycle consisting of denaturation at 94°C for 30 s, annealing at 57°C for 30 s, and extension at 72°C for 1 min and 15 s, with an initial denaturation at 94°C for 2 min and a final extension at 72°C for 5 min. Amplicons obtained were either gel or PCR purified and sequenced using standard Sanger technology. The sequences obtained were compared to the WT *FKB1* gene of *M. sympodialis* ATCC 42132 to identify genetic changes. Translation of the FKBP12 predicted proteins resulting from mutated *FKB1* genes was carried out using the online tool translator fr33 (http://www.fr33.net/translator.php), and translation of all the resulting sequences were subjected to multiple alignment using the online software Clustal Omega (https://www.ebi.ac.uk/Tools/msa/clustalo/). The same software was also used to generate a multiple alignment of the human FKBP12 (GenBank accession number AIC61966) with the fungal FKBP12 retrieved in previous analysis. Structural elements of the FKBP12 protein were indicated following a previous study of Tonthat and colleagues (37).

### Modeling the calcineurin-FKBP12-FK506 complex.

The *M. sympodialis* calcineurin subunits A and B, Cna1 and Cnb1, respectively, were retrieved from GenBank following BLASTp analysis using the *S. cerevisiae* Cna1 and Cnb1 orthologs. The SWISS-MODEL was used to build three-dimensional (3D) protein structural models using the crystal structure of the *Coccidioides immitis* FK506-inhibited calcineurin complex (Seattle Structural Genomics Center for Infectious Disease [SSGCID] et al., unpublished; SWISS-MODEL template library [SMTL] identifier [ID] or accession number 5b8i.1).

### Western blot analysis.

For protein extraction, cells of *M. furfur* CBS 14141 and *M. sympodialis* ATCC 42132 WT strains were collected from mDixon agar plates, while those of the respective *fkb1*Δ mutants and the independent spontaneous drug-resistant isolates of *M. sympodialis* were collected from mDixon agar plates containing 13 mM LiCl and 25 µg/ml of FK506. In all cases, strains were incubated for 3 days at 37°C prior to extraction, which was carried out as follows. Cells were resuspended in lysis buffer (10 mM Tris-HCl [pH 8.1], 150 mM NaCl_2_, 0.5 mM EDTA, 1% Triton X-100) containing protease inhibitors. Glass beads were added to the cell suspensions, and the cells were bead beat 20 times in 90-s intervals with 90-s rest between pulses. Lysates were cleared by centrifugation at 7,000 × *g* for 15 min at 4°C. Protein concentration was determined using Bradford reagent. Lysates (75 μg) were loaded onto a 10% Tris-glycine polyacrylamide gel and run at 120 V for 2 h. Proteins were transferred to a polyvinylidene difluoride (PVDF) membrane by wet electrotransfer (400 mA for 2 h) at 4°C. After blocking with 10% milk solution, blots were incubated with the anti-FKBP12 primary antibody overnight at 4°C. After incubation with the primary antibody, the blot was briefly washed with TST (10 mM Tris, 150 mM NaCl, 0.1% Tween 20) and then incubated with an anti-rabbit secondary antibody for 1 h at room temperature in a 5% milk solution. The blot was washed again three times with TST and exposed to an X-ray film by electrochemiluminescence. Loading control was done by staining with India ink for 1 h and destaining with numerous washes of phosphate-buffered saline (PBS).

### Mutation rate assays in the wild type and an *msh2*Δ mutant of *M. sympodialis*.

For qualitative assays aiming at assessing the FK506 resistance rate in the WT *M. sympodialis* ATCC 42132 strain and the derived *msh2*Δ mutant, four independent colonies of each strain were resuspended in 200 μl of liquid mDixon medium and then swabbed onto mDixon agar plus 15 mM LiCl with FK506 (25 μg/ml) or without FK506; the plates were incubated at 37°C for 5 to 8 days. For classical fluctuation assays, 10 independent colonies of the WT *M. sympodialis* ATCC 42132 strain and the derived *msh2*Δ mutant were grown for 48 h in 5 ml of liquid mDixon cultures at 30°C. The cells were pelleted, washed with distilled water (dH_2_O), and resuspended in 5 ml dH_2_O, and 1:10 serial dilutions were performed. One hundred microliters of a 10^−5^ dilution was plated on solid mDixon plus 15 mM LiCl, 100 μl of undiluted culture was plated onto solid mDixon plus 15 mM LiCl containing FK506 (25 μg/ml), and the plates were incubated at 37°C until growth could be recorded. Colonies that appeared in all types of media were counted, and the FK506 resistance rate was expressed as fold change ± standard deviation. For each strain, a single FK506-resistant colony derived from each independent replicate (i.e., 10 colonies per strain) was subjected to *FKB1* gene sequencing analysis as described before, with the only difference that the online software program T-Coffee (https://www.ebi.ac.uk/Tools/msa/tcoffee/) was used for protein alignment.
