# Erratum for Ianiri et al., “FKBP12-Dependent Inhibition of Calcineurin Mediates Immunosuppressive Antifungal Drug Action in *Malassezia*”

**DOI:** 10.1128/mBio.02055-17

**Published:** 2017-12-05

**Authors:** Giuseppe Ianiri, Shelly Applen Clancey, Soo Chan Lee, Joseph Heitman

**Affiliations:** a Department of Molecular Genetics and Microbiology, Duke University Medical Center, Durham, North Carolina, USA; b Department of Biology, South Texas Center for Emerging Infectious Diseases (STCEID), The University of Texas at San Antonio, San Antonio, Texas, USA

## ERRATUM

Volume 8, no. 5, e01752-17, 2017, https://doi.org/10.1128/mBio.01752-17. In describing structural models for three spontaneous FK506-resistant mutants shown in Fig. 7B (SR3, SR5, and SR6), the associated text incorrectly described these as mutations of lysine rather than leucine residues. The models shown are all correct, and this error does not alter the conclusion that these mutations are predicted to affect either FKBP12 conformation or stability to result in the observed drug resistance phenotype.

