# Supplementary figures and images for: FKBP12-Dependent Inhibition of Calcineurin Mediates Immunosuppressive Antifungal Drug Action in Malassezia
Source: mBio. 2017 Oct 24;8(5):e01752-17. doi: 10.1128/mBio.01752-17 (PMC5654937; doi:10.1128/mBio.01752-17)

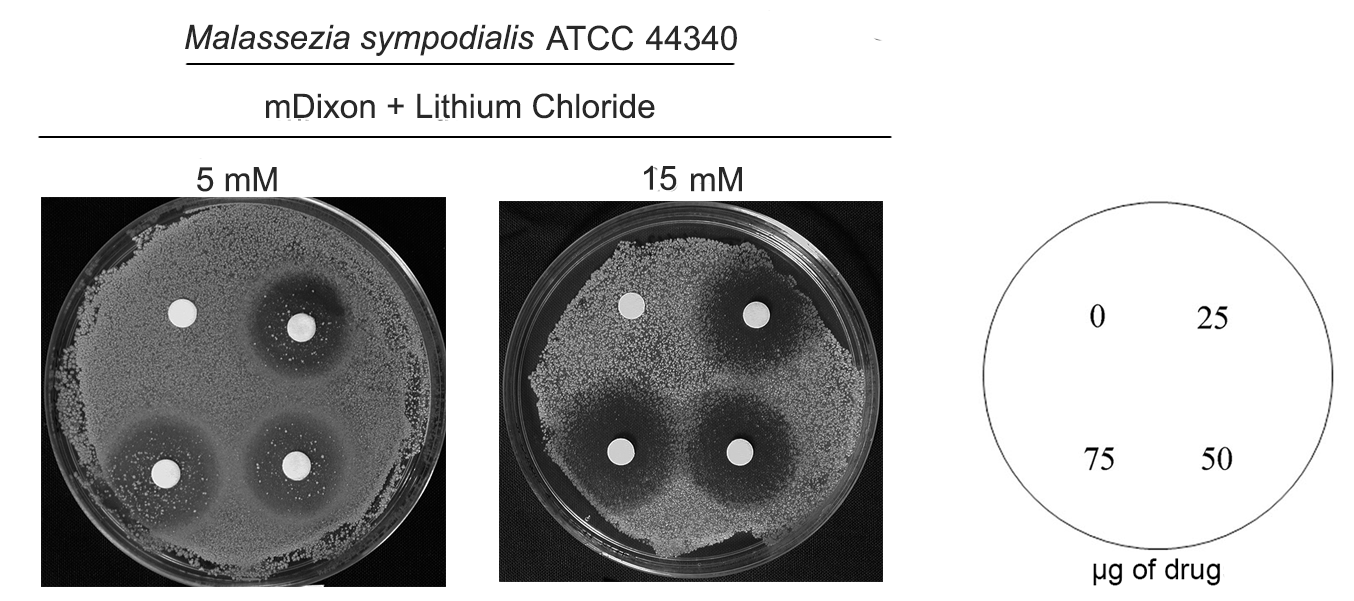

Supplement: FIG S1 [file mbo005173562sf1.tif]

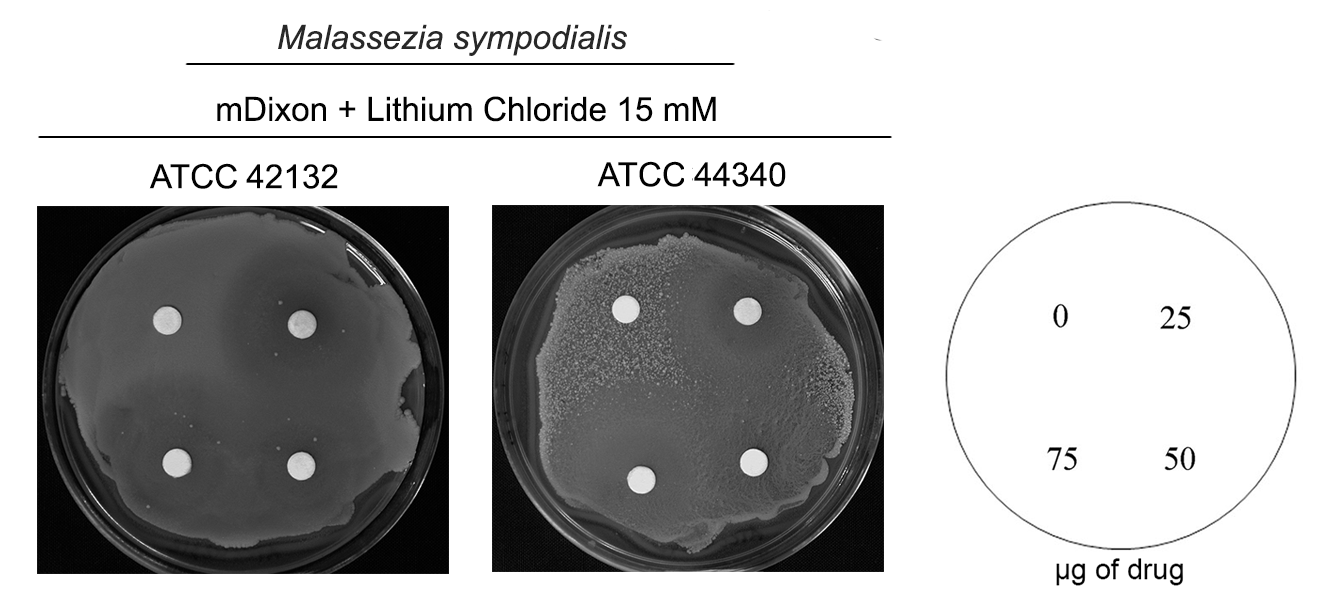

Supplement: FIG S2 [file mbo005173562sf2.tif]

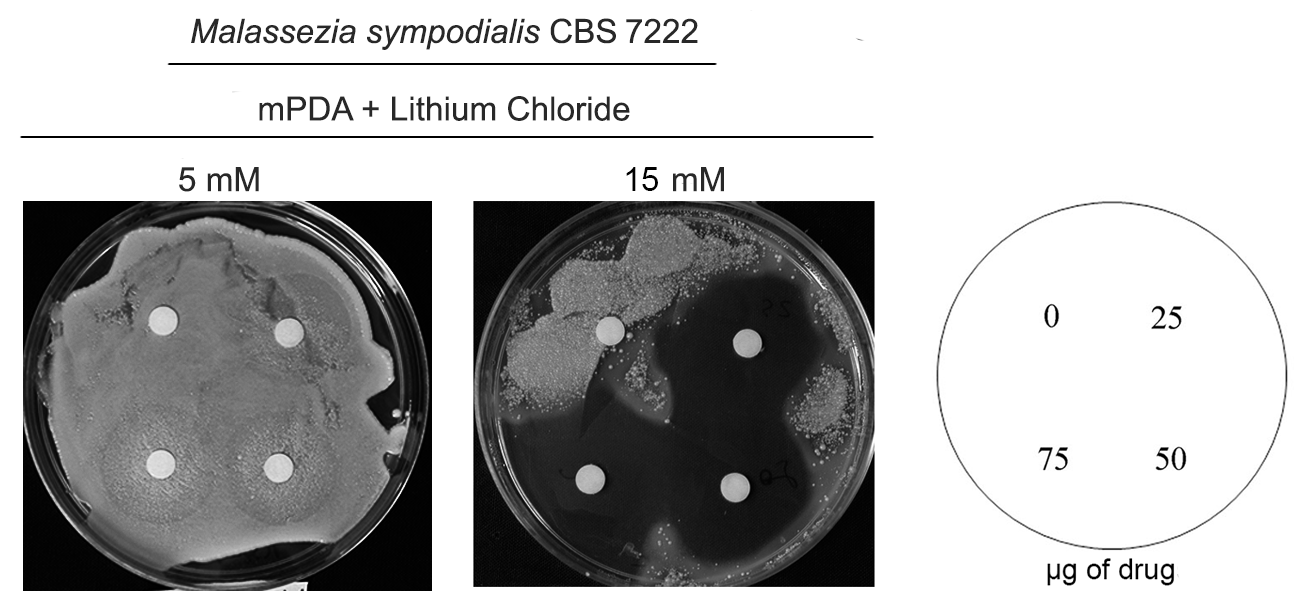

Supplement: FIG S3 [file mbo005173562sf3.tif]

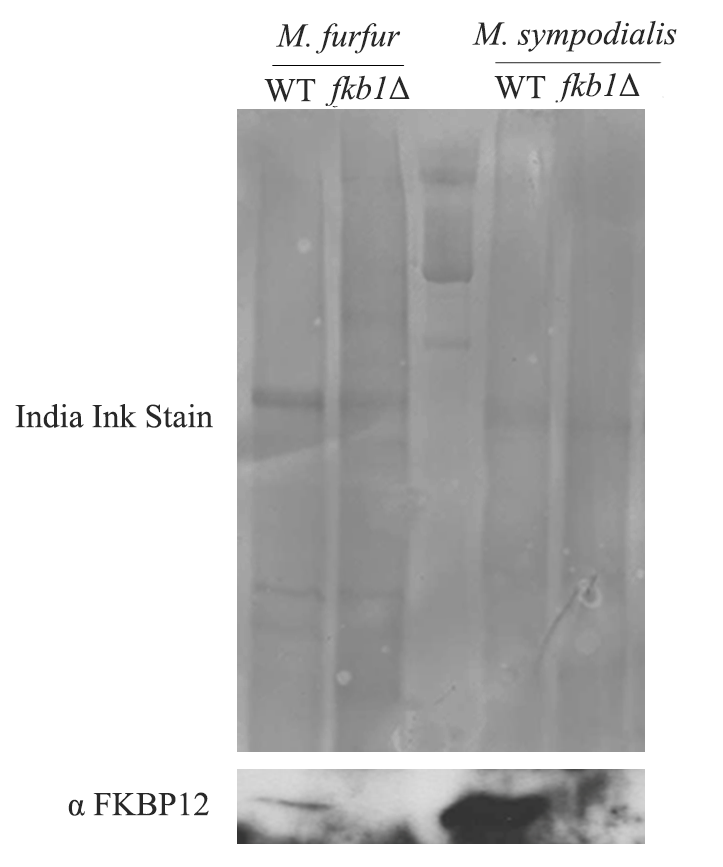

Supplement: FIG S4 [file mbo005173562sf4.tif]

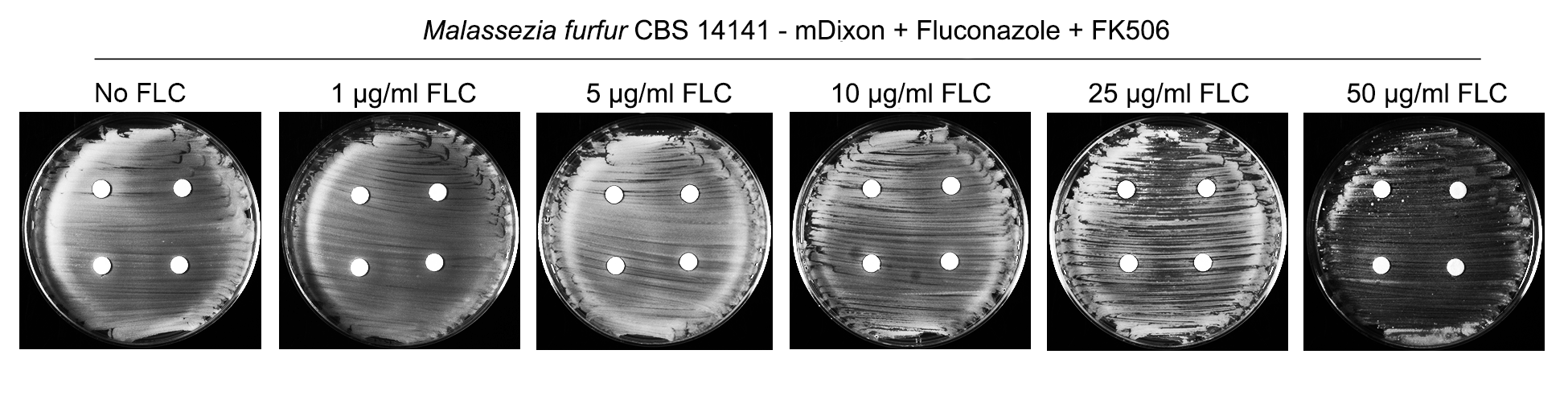

Supplement: FIG S5 [file mbo005173562sf5.tif]

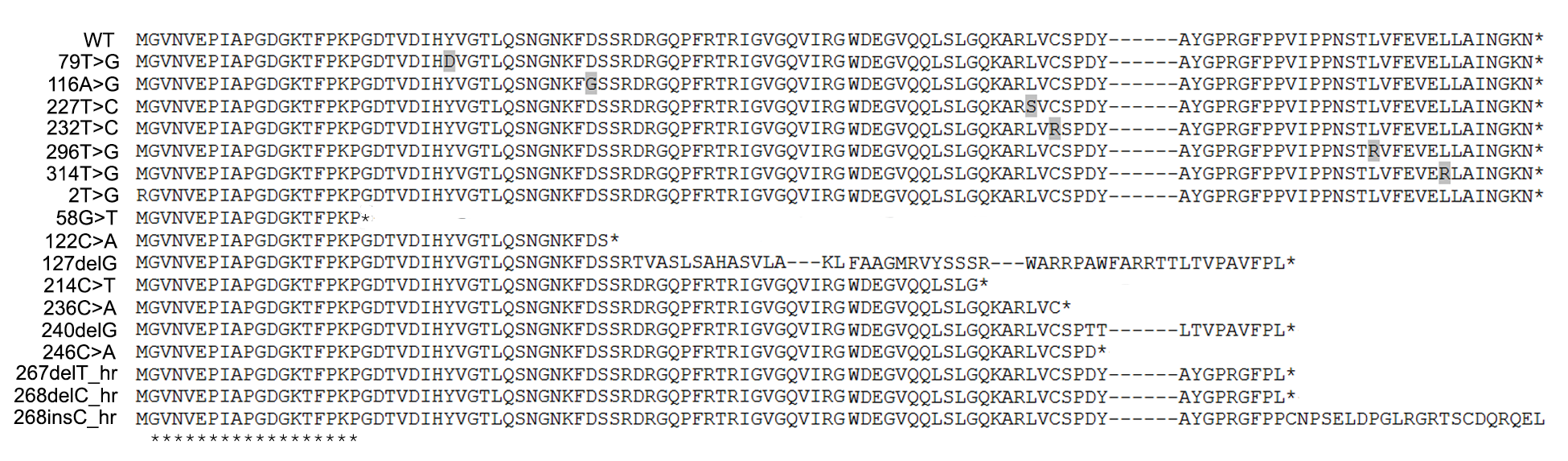

Supplement: FIG S6 [file mbo005173562sf6.tif]

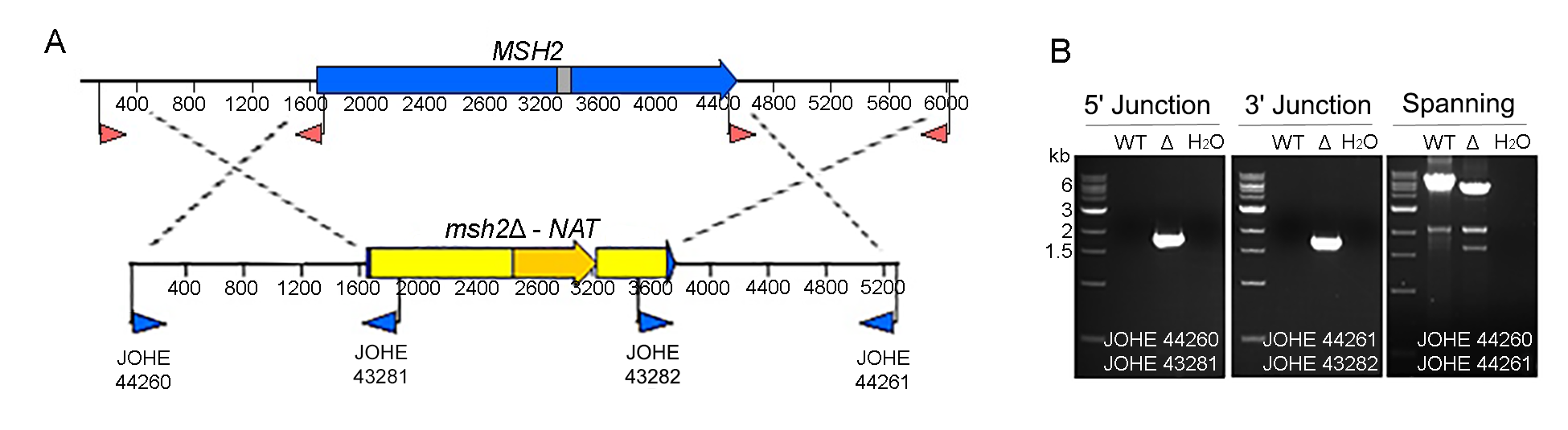

Supplement: FIG S7 [file mbo005173562sf7.tif]
